# Designing Patient-Friendly Messages: Tutorial on Applying Human-Centered, Self-Determination Theory With AI Considerations

**DOI:** 10.2196/78173

**Published:** 2025-10-17

**Authors:** Ashley C Griffin, Sarah J Javier, Madeleine Golding, Travis W Runnels, Marianne S Matthias, Stephanie L Shimada, Diana M Higgins, Donna M Zulman, Amanda M Midboe

**Affiliations:** 1 Center for Innovation to Implementation VA Palo Alto Health Care System Menlo Park, CA United States; 2 Center for Biomedical Informatics Research School of Medicine Stanford University Stanford, CA United States; 3 School of Medicine Stanford University Stanford, CA United States; 4 Enterprise Measurement and Design Veterans Experience Office United States Department of Veterans Affairs Menlo Park, CA United States; 5 Center for Health Information and Communication Richard L. Roudebush VA Medical Center Indianapolis, IN United States; 6 Regenstrief Institute Indianapolis, IN United States; 7 Department of Medicine School of Medicine Indiana University Indianapolis, IN United States; 8 Center for Health Optimization and Implementation Research VA Bedford Healthcare System Bedford, MA United States; 9 Department of Health Law, Policy, and Management School of Public Health Boston University Boston, MA United States; 10 Department of Population and Quantitative Health Sciences University of Massachusetts Chan Medical School Worcester, MA United States; 11 Durham VA Health Care System Durham, NC United States; 12 Department of Psychiatry Chobanian and Avedisian School of Medicine Boston University Boston, MA United States; 13 Division of Primary Care and Population Health, Department of Medicine School of Medicine Stanford University Stanford, CA United States; 14 Department of Public Health Sciences, Division of Health Policy and Management School of Medicine University of California, Davis Davis, CA United States

**Keywords:** patient participation, veteran, human-centered design, health behavior, mobile health, SDT, self-determination theory, artificial intelligence

## Abstract

Patient messaging technologies offer treatment information and recommendations through web-based platforms, patient portals, mobile apps, and SMS text messaging. Many of these technologies have started to incorporate messages that are crafted by artificial intelligence (AI). Such tools are most effective when constructed with theoretical grounding and iterative input from end users. Thus, we outline a human-centered design approach for developing patient messaging content that aligns with self-determination theory (SDT), a widely used framework that has shown positive impacts on health behavior change. We illustrate our approach step-by-step for the development of messages that promote evidence-based treatment opportunities for patients with chronic pain. Messages were initially developed by subject matter experts and refined using SDT constructs (autonomy, competence, and relatedness) and motivation and behavior change techniques. Using a rapid prototyping approach, we sequentially met with 3 patient engagement boards to elicit feedback on message prototypes and enhance their content. We synthesized and aligned disparate feedback across boards with SDT and motivation and behavior change techniques. Drawing upon the input from the engagement boards, existing co-design approaches, and the field of human-centered AI, we recommend strategies to collaborate with patient partners to enhance the readability and clarity of messaging content. Recommended strategies include (1) involve engagement boards early in messaging framing and modality selection, (2) represent diverse perspectives when refining messages, (3) acknowledge and set expectations to integrate unique experiences and views, (4) prioritize message tailoring for the population of interest, (5) incorporate continual feedback mechanisms, and (6) keep the human interaction in patient-facing messages. By illuminating the process of developing message content that aligns with SDT constructs and providing guidance for iterative patient engagement and practical prototyping, we hope this tutorial can be used to enhance patient messaging content and improve uptake of evidence-based treatments. Our approach and recommendations can also guide multidisciplinary research and design teams to build patient-centered health messages. This tutorial has special consideration for future AI-guided messaging interventions, as patients are typically not involved in message content development or framing, but early engagement can potentially mitigate known AI concerns related to privacy, transparency, and fairness. As technologies and patient populations change over time, linking continual end user input with theoretical grounding plays a key role in simplifying complex medical information and promoting understanding of treatment opportunities that can ultimately improve health outcomes.

## Introduction

### Widespread and Evolving Patient Messaging Technologies

Patient-facing health information technologies have dramatically increased in the last decade with the proliferation of smartphones and connected devices. Estimates indicate that 97% of US adults own a mobile phone of some kind [1], and phone-based messaging tools are commonly used to provide patient education and improve health behaviors [2]. This is, in part, because messaging tools, such as SMS text messaging, are convenient, low cost, and accessible for those with limited technology experience [2].

With the emergence of large language model (LLM) tools (eg, ChatGPT, Gemini, and Llama), which use enormous datasets to respond to inquiries, there is a growing focus on developing patient messaging tools to make information more accessible and improve health outcomes. These artificial intelligence (AI) products have demonstrated early potential to enhance patient education about procedures [3,4] and their understanding of discharge summaries [5]. Such tools may also be capable of providing patient-friendly information about evidence-based treatment options. Yet, the process of engaging in treatment often depends on how the message is communicated and can be improved with theoretical grounding and iterative input from patients, both of which are not typically involved in the design of AI-generated messaging. As connected health technologies increase, including the integration of AI, theory-informed techniques that facilitate patient engagement and feedback on the messaging content are critical to support effective interventions.

### Self-Determination Theory to Inform Health Interventions

The most sustainable health interventions are grounded in theories that account for patient motivations to engage in health behaviors and complex interactions between patients, clinicians, and health care systems [6]. One such framework is self-determination theory (SDT) [7,8], which has been applied extensively across health interventions. This theory centers around 3 basic psychological needs: autonomy (the ability to make one’s own decisions), competence (the ability to do something successfully or efficiently), and relatedness (feeling understood and cared for by others). It assumes that everyone’s capacity for behavioral change depends on whether these psychological needs are met. Importantly, SDT differentiates between internalized or intrinsic motivation and externalized or extrinsic motivation. It posits that behavioral change is more likely to occur and be sustained if individuals perceive that a change is consistent with their intrinsic values and goals. For instance, patients who perceive that their clinicians and health care systems are supportive of these needs being met experience greater intrinsic motivation to initiate and maintain health behaviors, such as adherence to treatment plans.

Interventions that promote psychological needs outlined in SDT have been shown to improve patient outcomes. In a meta-analysis of SDT studies, interventions that fostered autonomy, competence, and relatedness were moderately to strongly associated with better mental health, physical health, and medication adherence [9]. Given these positive outcomes, researchers have attempted to identify key components of SDT interventions that lead to motivation and health behavior change. One promising application of SDT is the development of patient-facing messages that enhance patients’ intrinsic motivation and autonomy to engage in certain healthy behaviors. Early evidence suggests that health-related messages framed in this way may lead to positive health behaviors, including smoking cessation [10,11], increased physical activity [12,13], and healthy eating [14]. Yet, there is little guidance on developing patient-facing messages that comprehensively address all SDT constructs, including how to develop the content and prototypes of messages guided by SDT.

### Patient Engagement in Human-Centered Design

Often complementing theoretical approaches, human-centered design [15] and related design disciplines (ie, user-centered design and design thinking) are commonly used to create and evaluate health interventions. These collaborative methods have been linked to improved patient acceptance and knowledge across a wide array of populations and health conditions globally [16]. Throughout the design process, patients and caregivers are often involved in providing input on their needs and during user testing and evaluation. Rapid prototyping, in particular, is an effective design approach for gathering insights and feedback on early prototypes, followed by refinement until the design satisfies user needs and requirements [17].

Patient engagement boards, which are representative groups of patients and caregivers who routinely provide input to improve processes and care [18], often have iterative longitudinal interactions with research, clinical, and design teams. They are also referred to as patient advisory panels, committees, and councils (hereafter referenced as “engagement boards”). Members of engagement boards are not considered “subjects” or “study participants,” but instead are key partners in the design process. Estimates indicate that approximately half of US hospitals have an engagement board [19], but few boards are involved in the co-design process [20]. Engagement boards are well-suited for designing patient messaging technologies, as patients and caregivers can be partnered with over time for co-building, learning, and continuous feedback. For instance, in one study, an engagement board was created among patients who had undergone cardiac surgery and their caregivers to inform the design and content of a mobile app to support surgery recovery [21]. Patients and caregivers attended 3 in-person sessions, where they discussed their perioperative journey, provided input on their informational needs, and gave feedback on a demonstration of the app. Collectively, this input culminated in a patient-centered app informed by lived experiences.

### Objective

In this tutorial, we outline key steps to achieve the following objectives: (1) develop messages aligned with SDT constructs that promote uptake of evidence-based pain treatments among patients with chronic pain at the Veterans Health Administration (VA) and (2) synthesize feedback from 3 patient engagement boards using human-centered design methods and further refine messages for optimal reach. We conclude by providing recommendations to guide multidisciplinary research and design teams to create meaningful health messaging content, particularly for AI-based messaging.

## Methods

### Ethical Considerations

This study was reviewed and approved by the institutional review board of Stanford University (#66202). Members of VA engagement boards are considered advisors rather than participants and are not required to be consented. Engagement board members received a stipend or voucher, which was paid by the VA, for participation. All study material was stored in a secure location to ensure privacy and confidentiality.

### Development of SDT-Based Messages

#### Overview of Steps 1-3

To meet our first objective, we partnered with VA clinical and health research psychologists with pain experience and researchers with extensive pain expertise (DMH, MSM, and AMM) who had previously developed messages to support pain self-management. We adapted these messages to focus specifically on 3 nonpharmacological psychological and behavioral treatments for chronic pain, which included cognitive behavioral therapy, mindfulness-based stress reduction, and acceptance and commitment therapy. Nonpharmacological approaches are recommended as the first line of treatment for chronic pain in the VA; yet, these evidence-based treatments have historically had low rates of patient engagement [22,23]. We also conducted a literature review to ensure that the psychological and behavioral pain treatment messages reflected constructs from SDT. Finally, we developed message prototypes to visually represent common technology modalities, where patients could view the messages.

#### Step 1: Initial Message Development

**Figure 1:**
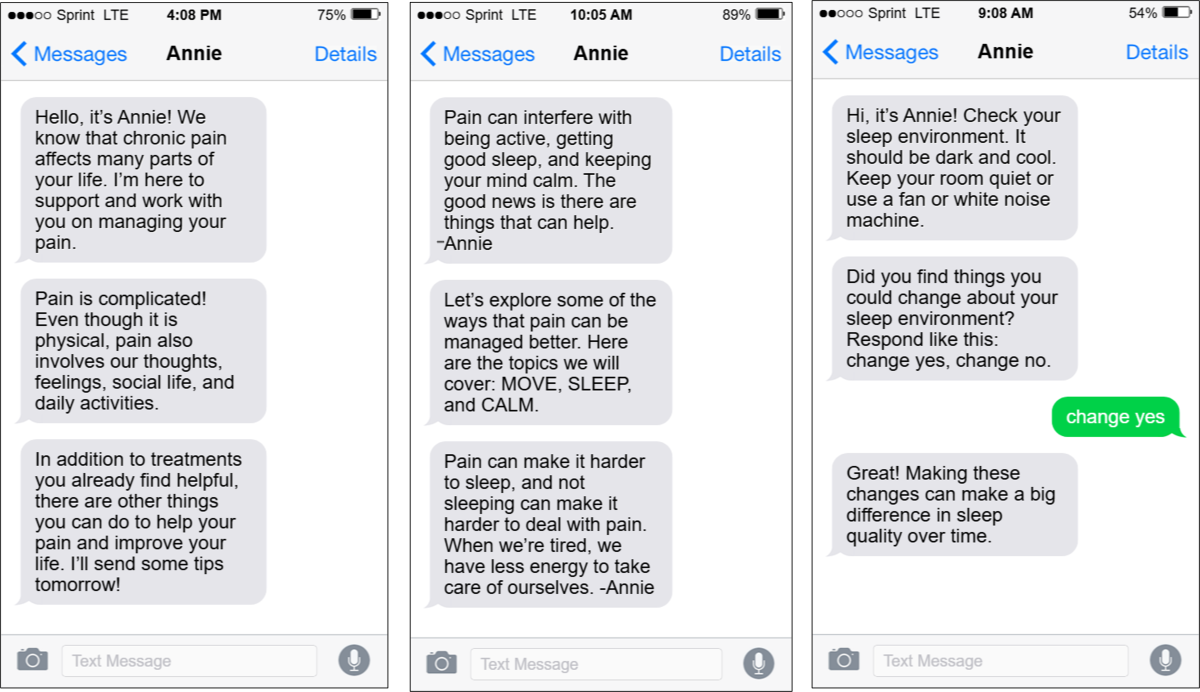
Example messages to promote awareness of nonpharmacological pain management strategies that were initially developed for the Veterans Health Administration’s Annie SMS text messaging service.

Prior to this project, the VA Pain Management, Opioid Safety, and Prescription Drug Monitoring Program Office tasked the aforementioned VA subject matter experts with developing short SMS text messages about pain management approaches, including topics on sleep hygiene, mindfulness, and self-care. These texts were formatted for deployment via Annie, a VA SMS text messaging service that sends automated messages to veteran patients [24]. Messages were iteratively refined by both subject matter experts and an Annie expert (Figure 1). The messages developed in step 1 served as the foundation for the messages in step 2.

#### Step 2: Theory-Driven Message Refinement Using SDT and Motivation and Behavior Change Techniques

In this step, we refined messages to focus on evidence-based psychological and behavioral treatments, as opposed to general pain management strategies. ACG and SJJ first conducted a literature review of health interventions enacting components of SDT, including autonomy, relatedness, and competence. Teixeira et al [25] developed a list of 21 motivation and behavior change techniques (MBCTs) linked to SDT via an iterative expert panel consensus study. These techniques include specific ways to bolster components of SDT, including autonomy (eg, use noncontrolling, informational language), relatedness (eg, acknowledge and respect perspectives and feelings), and competence (eg, help develop a clear and concrete plan of action). For example, a technique to bolster autonomy support is using nonjudgmental language that conveys choice, such as using “might” or “could” instead of “should.” Following the literature review, ACG and SJJ engaged in iterative development and refinement of messages, with concurrent review by a team of subject matter experts in chronic pain, psychology, and behavioral health (DMH, MSM, and AMM) during each iteration (see Multimedia Appendix 1 for the final set of messages [25]).

#### Step 3: Creation of Digital Prototypes

**Figure 2:**
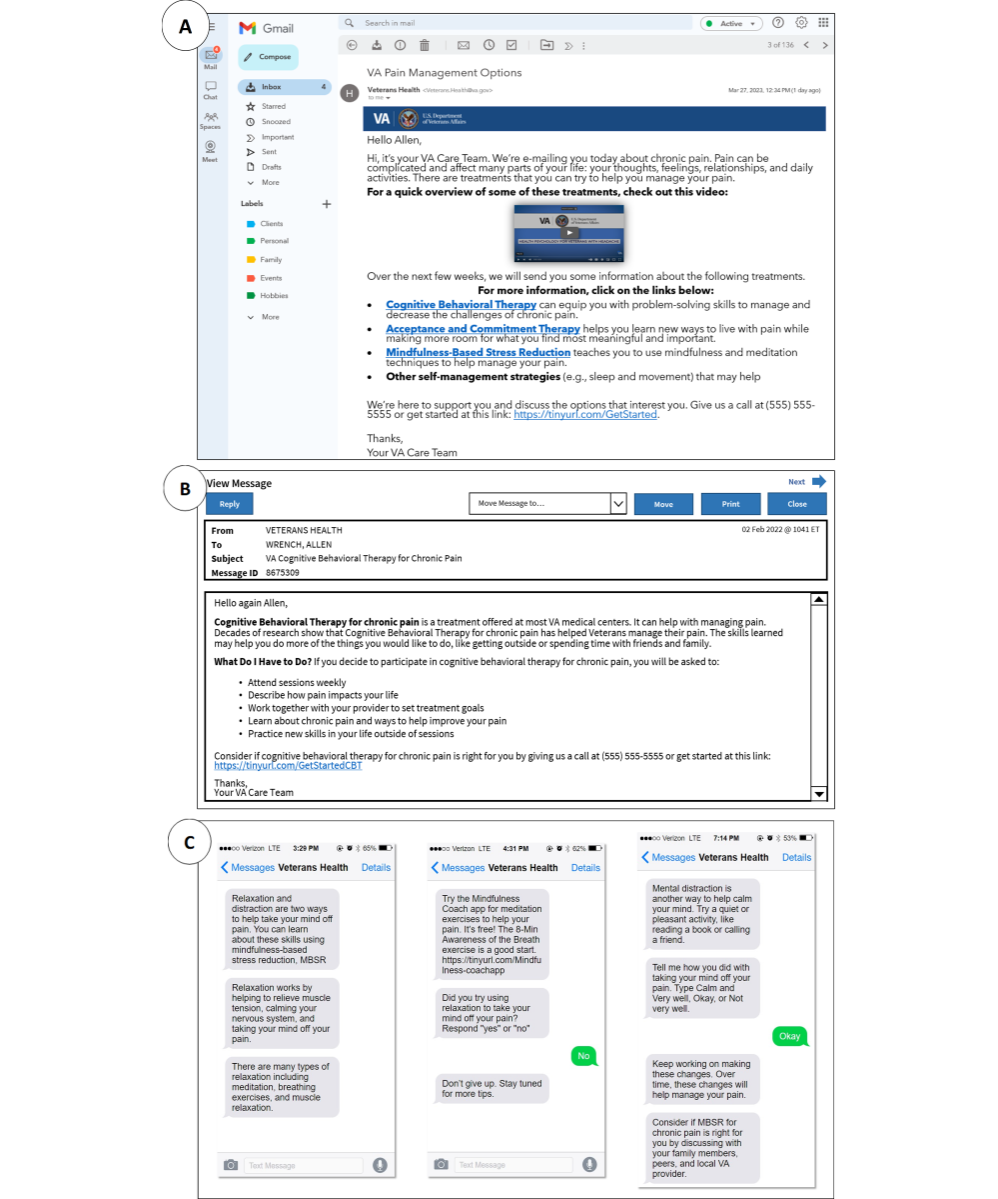
Examples of high-fidelity pain care message prototypes generated for patient engagement board review. (A) Introductory email message prototype depicting an overview of 3 behavioral treatments and other self-management strategies. (B) My HealtheVet patient portal secure message prototype containing a description of cognitive behavioral therapy. (C) Text message prototypes with information about mindfulness-based stress reduction and the ability for patients to respond.

Prototypes are tangible examples of abstract ideas that can be used to generate knowledge and feedback [26]. It was important to develop prototypes that visually represented common technological modalities to give engagement boards realistic examples to react to. As such, we created digital prototypes for messages that would appear on various patient-facing platforms: email, patient portal message, and SMS text message (Figure 2). We used free internet resources (eg, iFake Text Message) and replicated email and patient portal interfaces on Microsoft PowerPoint to develop prototypes.

### Consulting Patient End Users for Feedback Using Human-Centered Design

#### Overview of Steps 4-6

To meet our second objective, we sequentially met with patients and caregivers on 3 patient engagement boards and used rapid prototyping after each meeting to improve the content and understanding of the messages between iterations. We found it beneficial to involve engagement boards both early in the design process and often thereafter, as it allowed us to create a more targeted set of messages informed by end users.

#### Step 4: Ensure Adequate Representation of End Users on Engagement Boards

Patients, families, and caregivers participating on engagement boards should be representative of the target population and include diverse backgrounds, expertise, and experiences. We sought to have a variety of chronic pain experiences represented, including patients with opioid use disorder, as it is common among patients with chronic pain [27]. As such, we identified 3 separate engagement boards of varying demographics: Veteran and Family Advisory Committee [28], Pain/Opioid Consortium of Research Veteran Engagement Panel [29], and Substance Addiction and Recovery Veteran Engagement Board (formerly Opioid Addiction and Recovery Veteran Engagement Board) [30]. Each board is comprised of approximately 13-18 veterans or family members of diverse demographics and backgrounds. The latter 2 boards focus on the input of patients from across the United States with a lived experience of chronic pain, opioid medication use, or opioid use disorder.

We found that patients’ input varied based on their backgrounds and experiences with pain and prior treatments. Some words and phrases that seemed to be beneficial in initial drafts deterred some patients. As one example, some patients pointed out that phrases including “relax” may not be effective for someone who is experiencing anxiety because of their pain. Our team had to weigh these differing opinions and feedback, which is a common issue during the design process. We sought to align contrasting feedback with SDT constructs and the MBCTs (see step 6).

#### Step 5: Rapid Prototyping Between Patient Engagement Board Meetings

**Figure 3:**
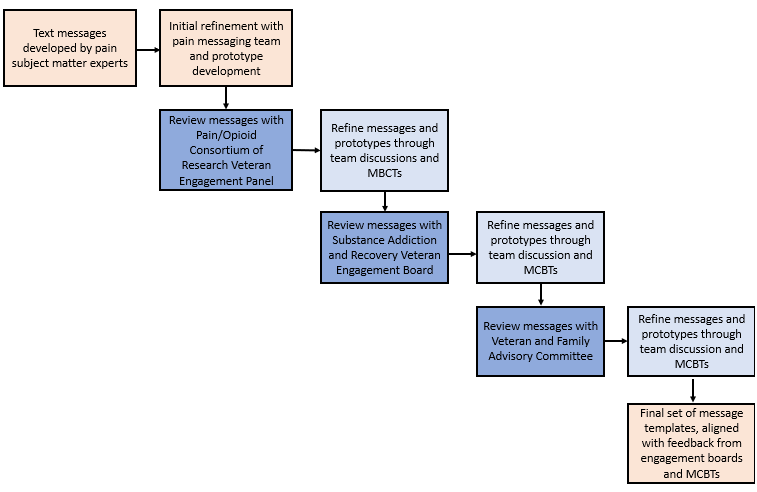
Overview of the rapid prototyping approach to refine pain care message content among 3 Veterans Health Administration engagement boards between December 2022 and January 2023. MBCT: motivation and behavior change technique.

To refine the messages, we incorporated feedback from each engagement board prior to meeting with a subsequent board using rapid prototyping (Figure 3). Before each meeting, our team was required to submit information and materials that we planned to present and specify the types of feedback requested from patients. These premeeting materials were crafted using lay terms so that all engagement board members would have the opportunity to provide feedback regardless of education levels. Researchers should account for the time required to prepare such premeeting information as well as be intentional and specific about the types of feedback desired. We also had to be flexible in terms of scheduling, as each engagement board had a set day each month dedicated to meeting with researchers.

During each engagement board meeting, patients were asked to reflect on what they liked and disliked about the prototypes, changes, and additions and what would be the most effective way to communicate this information to patients with chronic pain. It is important to note that each meeting was facilitated by a representative of the board who was often a veteran or patient. The presence of a meeting facilitator allowed the researchers (ACG and SJJ) to focus on feedback and engagement with board members while the facilitator took notes. After each meeting, ACG and SJJ refined prototypes through team-based discussions and consensus. We reviewed meeting transcripts, corroborated notes, and consulted with our larger team’s subject matter experts to align feedback with prototype edits.

#### Step 6: Synthesis of Patient Input and Prioritization Using Theory

**Figure 4:**
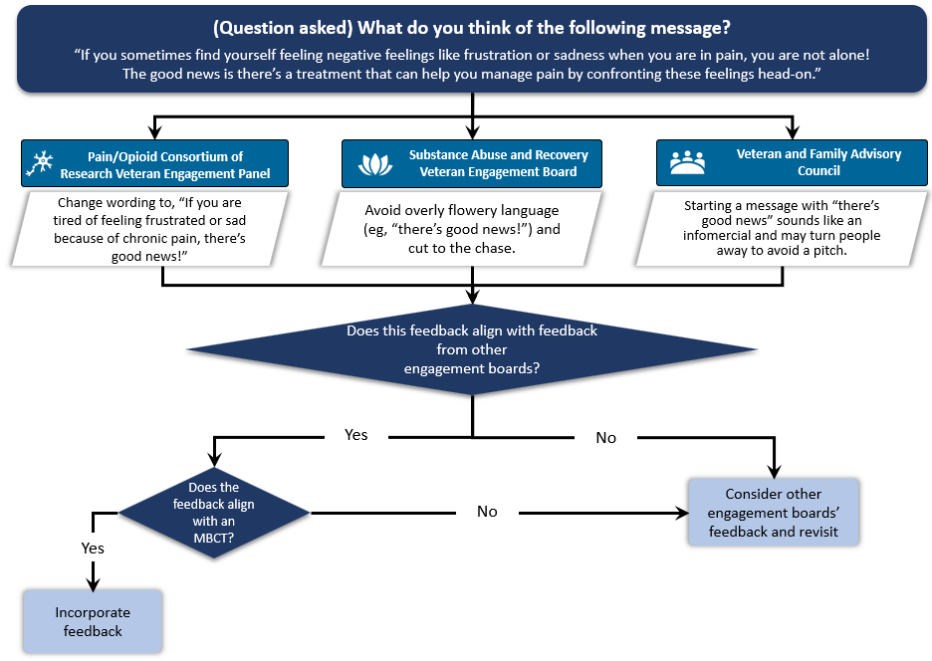
Decision process for aligning input from 3 Veterans Health Administration engagement boards with MBCTs linked to self-determination theory. MBCT: motivation and behavior change technique.

Overall, there was a lot of variation in patient preferences regarding the content and language of the messages, which differed both within and across engagement boards. Our decision process to compile input from each engagement board, synthesize feedback, and align decisions with the most related MBCT is depicted in Figure 4. For example, some patients recommended phrases such as “There’s good news!” while others believed that a message that started with this phrase would sound like an infomercial and turn people away. Leveraging MBCT 11 (Demonstrate and show interest in the person) and rearranging the message resulted in the following: “If you sometimes feel frustrated or sad because of your chronic pain, the VA offers Acceptance and Commitment Therapy which can help.”

**Figure 5:**
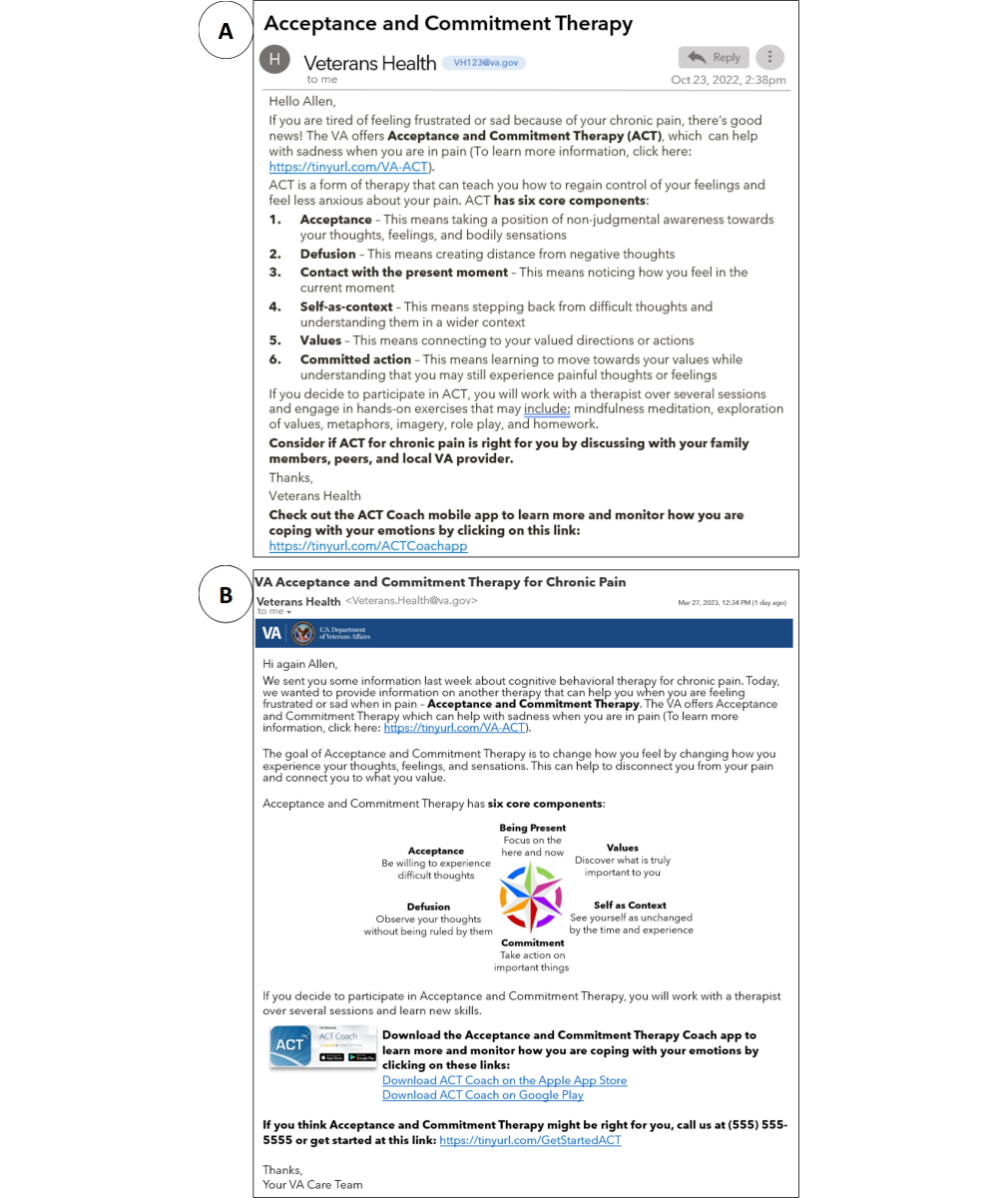
(A) Initial pain care message prototype and (B) final prototype of an email providing information on acceptance and commitment therapy, informed by input from 3 Veterans Health Administration engagement boards.

Patients also provided valuable feedback on setting expectations about the duration, frequency, and sender of the messages. Some recommended specifying the period of messages (“Over the next two weeks you’ll receive messages about pain treatments”), and others were interested in knowing the number of messages (“Message 1 of 10”), as receiving too many messages might discourage patients from participating. Patients also commented on the importance of the first sentence in a message; some preferred to begin with “We’re e-mailing you about your chronic pain,” while a few desired a statement that resonated with them such as “Pain is complicated.” To be inclusive of varying views, we framed the introductory messages based on the MBCTs for clarifying expectations (MBCT 16) and providing statements that acknowledge and respect perspectives and feelings (MBCT 8). For instance, our final refined introductory message began with: “Hi, it’s your VA care team. We’re e-mailing you about chronic pain. Pain can be complicated and affect many parts of your life ... Over the next few weeks, we will send you some information about the following treatments.” Figure 5 provides a comparison of the initial prototype and final prototype message for acceptance and commitment therapy after synthesizing engagement board input and alignment with MBCTs (see Multimedia Appendix 2 for additional message prototypes).

## Discussion

### Summary of Designing Patient-Friendly Messaging Content

This tutorial provides an approach for designing the content of patient-facing messages with prioritization of diverse feedback from patient engagement boards. We focus on aligning messages with SDT and gathering iterative feedback from patient end users through rapid prototyping. We illustrate this approach for the development of messages (ie, email, SMS text, and patient portal) that promote awareness of evidence-based pain treatments among patients in the VA. This work can serve as a step-by-step guide for multidisciplinary researchers, designers, and developers to build patient-centered messages.

This work is aligned with existing frameworks and guides focused on integrating behavioral theories into the human-centered design process [31-34]. We extend prior work by providing guidance for the development of messaging content that aligns with SDT constructs through iterative patient engagement and practical prototyping. SDT has been gaining traction in the design and human-computer interaction communities to enhance user motivation of healthy behaviors [35,36]. However, to our knowledge, there is little guidance for aligning messaging content with patient input and SDT. As design teams are often faced with difficult decisions in weighing differing input, this tutorial provides a unique lens on compiling varying perspectives to develop message content.

Our approach focuses on human-generated messages from subject matter experts, as we prioritized accurate, appropriate, and high-quality content. While generative AI is transforming how messaging content is created, it is unknown whether AI-generated messages would be as effective as human-generated messages. Early studies comparing human-generated messages to AI-generated messages have found that AI messages are generally evaluated as acceptable and can be perceived as more persuasive than human-generated messages [37-39]. For instance, in a study comparing individuals’ perceptions of the Centers for Disease Control and Prevention COVID-19 provaccination messages to messages generated by OpenAI’s GPT-3, the GPT-3 messages were perceived as more effective and having stronger arguments than the Centers for Disease Control and Prevention messages [39]. However, individuals preferred public health messages originating from human organizations rather than AI sources [39]. Other comparative work has also cited concerns with AI-generated messages containing false statements [37] and a lack of cultural relevance for certain populations [40]. Checking message content for accuracy if AI is used to create the initial messages (step 1) is important to mitigate AI hallucinations or erroneous claims of effectiveness to make the messages more convincing.

### Recommendations for Partnering With Patient Advisors to Enhance Messaging Content

#### Overview of Recommendations

Based on the input received from the engagement boards and our teams’ reflections, we identified strategies to collaborate with patient partners to enhance health messaging content. Our recommendations draw upon guiding principles and lessons learned from patient engagement literature [18,41,42], co-design approaches [43-45], and the field of human-centered AI [46]. We provide examples based on our messaging approach for patients with chronic pain, which has considerations for other patient populations and emerging AI-based messaging. By reflecting on this process, we hope these recommendations can be used to enhance the comprehension and clarity of patient-facing messages to improve uptake of evidence-based treatments.

#### Recommendation 1: Involve Patient Engagement Boards Early in Message Framing and Modality Selection

We sought feedback on the messages to ensure that the content reflected appropriate framing, language, and accessible modalities for patients to learn about evidence-based pain treatments. At an early stage, we found it valuable to gather input on the clarity, readability, relevance, and tone of the messages. For instance, some patients preferred messages with a warmer tone, such as “your care team is thinking of you and wants to work with you,” whereas others recommended using language that grabs attention by providing the success rates of the treatment for alleviating pain. Patients also generally agreed that messages should be available through multiple modalities, including SMS text messaging, email, and patient portal, which have important early design considerations for the structure, brevity, character limit, subject line, and timing of messages.

For AI-based messaging, little work has been done to involve patients in their content or framing, but early engagement can potentially mitigate known AI concerns related to privacy, fairness, and transparency. A 2023 national survey of US adults found that most individuals have low trust in health systems to responsibly use AI [47]. This is to be expected with novel and unfamiliar technologies, as most US adults have a limited understanding of AI and are largely unaware of common uses of AI [48]. Prior work eliciting input from patients on AI health app development found that educating patients on AI foundations is vital to facilitate meaningful engagement in technology designs [49]. Any engagement board designing AI-related messages should receive training on AI.

#### Recommendation 2: Represent Diverse Perspectives When Refining Messages

We incorporated end users’ feedback throughout the message refinement process, emphasizing diversity across an array of characteristics throughout the engagement boards. Sociodemographic factors as well as education, rurality, clinical needs, and technology aspects (eg, digital literacy, accessibility, and manual dexterity) may affect the use of messaging interventions. For clinical needs, in addition to meeting with patients with chronic pain, we sought input from patients with histories of opioid or other substance use disorders. We found that these patients’ perspectives were especially helpful for crafting messages that would resonate with patients with chronic pain who were prescribed opioids or other patients with opioid use disorder. To promote digital inclusion and avoid widening the digital divide, thoughtful attention is needed to make engagement board participation as easy and appealing as possible to patients who may be less familiar with using technology or the research process. For instance, research teams should think about their work in a simplistic way and be able to translate it to lay terms when meeting with engagement boards, opening the ability for everyone to participate.

Diverse patient perspectives are especially valuable for AI-based messaging approaches, given the well-known algorithmic biases that lead to different outputs across patient populations [50]. This often results in fewer benefits for specific individuals or groups. For example, a study examining LLMs found that the models can recommend different medical treatments for the same condition based on a patient’s sociodemographic characteristics, particularly for race, housing status, and gender [51]. For the design of messages intended to promote uptake of evidence-based treatments, having appropriate representation is needed to avoid perpetuating existing biases that might deter some patients from trying a new treatment.

#### Recommendation 3: Acknowledge and Set Expectations for Integrating Unique Experiences and Views

It is essential to recognize that each patient has a unique experience and background and that their contributions are important for designing messaging interventions. Based on these distinct experiences, expectations should be set with engagement boards on how their feedback will be prioritized into messaging designs, such as on a certain criterion like alignment with theory, practicality, or accessibility. While it was important to have a final set of messages that reflected the rich feedback we received, we also wanted the final message set to align with SDT, given that interventions informed by this theory have been shown to have positive effects on health behavior change [9]. Other researchers and design teams will need to decide whether patient feedback supersedes theoretical alignment in their unique studies. At a minimum, teams should be intentional about how they are going to handle patient input when it does not align with the selected theory (see recommendation 4).

Researchers and design teams should also consider how curated messages that incorporate patient input and theory can serve as training data for AI-based messaging approaches. As this work focused on developing messages for 3 types of nonpharmacological pain treatments, future work could explore how generative AI could leverage our existing corpus to expand the messages to other evidence-based treatments for chronic pain or other conditions. Integrating patient input and feedback on such a hybrid set of messages would be a key step in evaluating the feasibility of this approach.

#### Recommendation 4: Prioritize Message Tailoring for the Population of Interest

For certain messages, we prioritized feedback that most closely aligned with MBCTs due to the empirical evidence of interventions based on SDT in altering health behaviors. We also prioritized feedback from the board whose members most closely resembled the patients for whom the intervention was designed (ie, patients with chronic pain). We received diverse feedback across the 3 engagement boards and had to make systematic decisions to finalize the messages. In an ideal world, we would design a system that could generate unique messages based on distinct patient characteristics, such as clinical, social, and technical factors. Some patients mentioned how rather than the message displaying “the treatment can help you do more of what you would like to do,” it should be personalized based on their unique goals, such as “the treatment can help you spend more time with your friends and family.”

With the advancement of LLMs, such data have the potential to be extracted from electronic health records and clinical notes to facilitate the personalization of interventions. However, a recent systematic review found that only 5% of studies are using LLMs with real patient data [52]. It is evident that these emerging tools warrant further study to understand patients’ perceptions and the implications of integration with their health data. This would also involve understanding patients’ concerns about using their data to train an algorithm, such as handling sensitive health information, unintended consequences, or ethical issues.

#### Recommendation 5: Incorporate Continual Feedback Mechanisms

Gathering iterative input and continual feedback from engagement boards can build bridges for future collaborative work with engagement boards [53]. Design teams should be flexible and open to suggestions for improvement on each iteration. For instance, we had the opportunity to have a follow-up meeting with 2 of the engagement boards. During this time, we presented the final messages and prototypes based partially on their feedback. We described how their insights were helpful for creating the messages and prototypes and to ensure they would resonate with patients with similar experiences. Patients on each board were positive about the changes we made and felt encouraged that their input and ideas were included. They also offered further suggestions to improve digital communication, outreach through caregivers, and health tools geared toward patients in recovery.

Prior work proposed a framework for integrating continual perspectives into AI language tools using a community-based participatory research approach [54], referred to as “community-based natural language processing.” The framework outlines ways to involve communities in developing AI health tools spanning the entire development pipeline, ranging from data curation to model deployment and validation. Such approaches could be used to routinely engage with patients over time to foster collaborative, meaningful AI messaging content.

#### Recommendation 6: Keep the Human Interaction in Patient-Facing Messages

In addition to the message content, several patients emphasized the importance of a human component in messages they receive. This is aligned with the SDT construct of relatedness, as it pertains to feeling understood and cared for by others. Moreover, patients felt that sometimes messages can lead to frustration if they are not interactive. This finding is concordant with the computers are social actors theorem, which describes how people communicate with computers as if they are people [55]. Prior work on this topic has also shown that frustrating interactions with computers can lead to strong negative emotions toward the system and its developers [56]. Some patients recommended that it would be more beneficial to use AI to answer certain types of questions rather than the system resending information. At a minimum, it was suggested to provide a way to get support from another person, including a phone number. This may be especially important for some groups of patients, such as those with opioid use disorder, who may need more frequent interactions with their care team.

Ensuring patient-care team relationships are not harmed by AI tools is critical, as there are concerns about the loss of the human element from these new technologies [48]. The human-centered AI field recently emerged to embrace a human-centered perspective on building AI tools to better serve human needs [46]. This approach builds on typical design methods of stakeholder engagement, user observation, testing, and iterative refinement for systems that use AI. Actively involving patients throughout the design and development process of AI tools emphasizes using AI to improve communication and empower patients and care teams, rather than replacing human interactions. Future efforts should examine effective combinations of hybrid AI-human messaging interventions, including when it is suitable for a human versus AI to respond to certain questions and when it is appropriate to connect the patient to the care team.

### Limitations

This effort was conducted within the VA health care system, which is a large integrated health system that has established several veteran engagement boards. Thus, our approach may not generalize to other health care systems with different levels of resources or to other patient populations. In addition, as with all studies involving group-based discussions, there is potential for some participants to have strong voices in the conversation as well as for conformance to socially acceptable views or group consensus. However, each engagement board meeting was led by a skilled moderator associated with the board who encouraged open dialogue based on each patient’s unique experience.

In addition, the focus of this tutorial was on messaging content, and other design elements should be considered when developing messaging interventions. For example, understanding how the messages fit into the patient’s routine, how patients interact with the messages over time, and workflows if patients respond to the messages should also be examined if the messages are to achieve the desired outcomes. Finally, this tutorial centered around SDT, but there are a number of other well-studied behavioral theories for understanding factors that influence health behaviors. Our approach has not been trialed with other theories to develop patient messaging content, but our recommendations do provide strategies for engaging with patients to develop messaging content that could apply to other theoretical frameworks.

### Conclusions

With the rapidly expanding patient messaging ecosystem and integration of AI, partnering with patient engagement boards over time offers the opportunity to cobuild and iterate on messaging content that is patient-friendly and safe. We found that patients with different experiences with chronic pain and backgrounds had varying reflections on the content and motivational techniques for trying new types of pain treatments. Anchoring patient input in SDT and the MBCTs assisted with synthesizing and prioritizing feedback on the messages. Patient input and our reflections resulted in the following recommendations to improve the design of patient messages: (1) involve patient engagement boards early in message framing and modality selection, (2) represent diverse perspectives when refining messages, (3) acknowledge and set expectations for integrating unique experiences and views, (4) prioritize message tailoring for the population of interest, (5) incorporate continual feedback mechanisms, and (6) keep the human interaction in patient-facing messages. As patients and technologies change over time, combining continual end user input with theory-based approaches is valuable to improve the comprehension and understanding of patient-facing messages for improved treatment opportunities and outcomes.

## Appendix

Multimedia Appendix 1 Nonpharmacological pain care messages mapped to motivation and behavior change techniques and self-determination theory constructs (autonomy, relatedness, and competence).

Multimedia Appendix 2 Pain care message prototypes for email, patient portal, and text messaging.

## Abbreviations

AI: artificial intelligence
LLM: large language model
MBCT: motivation and behavior change technique
SDT: self-determination theory
VA: Veterans Health Administration

## Data Availability

The data generated and analyzed during this project are not publicly available due to the sensitive information collected from patient engagement board meetings. Anonymous data are available from the corresponding author upon reasonable request, subject to institutional or regulatory approvals.
