# Supplementary material for: Designing Patient-Friendly Messages: Tutorial on Applying Human-Centered, Self-Determination Theory With AI Considerations
Source: J Med Internet Res. 2025 Oct 17;27:e78173. doi: 10.2196/78173 (PMC12579294; doi:10.2196/78173)
Supplement: Multimedia Appendix 1 [file jmir_v27i1e78173_app1.docx]

| **Motivation and Behavior Change Technique** | **Definition Adapted for**  **Current Project** | **Example of Pain Care Message** |
| --- | --- | --- |
| **Autonomy-Support Techniques** | | |
| 1. Elicit perspectives on condition or behavior | Encourage the patient to explore and share perspectives on their current pain management routine. | Do you sometimes find yourself feeling negative feelings like guilt, frustration, and sadness when you are in pain? You are not alone! |
| 2. Prompt identification of sources of pressure for behavior change | Prompt the patient to identify sources of external (or partially internalized) pressures and expectations to change pain management routine and explore how they relate to desired goals and outcomes. | Pain can make it harder to sleep, and not sleeping can make it harder to deal with pain. When we’re tired, we have less energy to take care of ourselves. |
| 3. Use non-controlling, informational language | Use informational, non-judgmental language that conveys freedom of choice, collaboration with pain care clinicians, and possibility when communicating. | Cognitive Behavioral Therapy for chronic pain can help address sleep concerns. One thing that you could check on is your sleep environment. Try making it dark and cool. Keep your room quiet or use a fan or white noise machine. |
| 4. Explore life aspirations and values | Support the patient in identifying important life aspirations, values, and long-term interests, and explore how changes in pain management routine vs. no changes could be linked to them. | It’s common to worry that movement will make your pain worse. But regular movement is actually helpful for managing your pain. |
| 5. Provide a meaningful rationale | Prompt the patient to identify rationale for changing pain management routine and seeking out information that is tailored, explanatory, and personally meaningful. | Pain can affect many parts of your life. Even though it is physical, pain also involves our thoughts, feelings, social life, and daily activities. The good news is there are things that can help. |
| 6. Provide choice | Provide opportunities to make choices from a menu of different evidence-based treatments for chronic pain and autonomous goals. Also provide choices for the way in which they receive information about these treatments. | Cognitive Behavioral Therapy for chronic pain treatment consists of an assessment session and 10 active, skills-based therapy sessions. You can choose to do these sessions in the clinic or from the comfort of your home with most VA clinicians. |
| 7. Encourage the person to experiment and self-initiate the behavior | Allow the patient to self-initiate information-seeking about psychosocial treatments for chronic pain in a way that is approachable for them and fosters positive reinforcement (e.g., providing links to informational videos, providing discussion prompts for discussing the treatments with clinicians). | You might also try creating a bedtime routine to help you wind down. For example, you could read, take a warm bath, or listen to quiet music. |
| **Relatedness-support techniques** | | |
| 8. Acknowledge and respect perspectives and feelings | Provide statements of empathy and acknowledgment of the patient’s perspective and both negative and positive emotions towards pain and pain care. | Pain is complicated! |
| 9. Encourage asking of questions | Prompt the patient to ask their care team about evidence-based psychosocial treatments for chronic pain. | We encourage you to ask questions and discuss the options that interest you with your VA care team. |
| 10. Show unconditional regard | Express positive support regardless of whether or not the patient seeks out information about psychosocial treatments for chronic pain. | Interactive Question: Did you think of ways to increase your movement throughout the day? Respond like this: *Increase yes*, *Increase no*.  *Increase yes*: Great! Making these changes can make a big difference in pain over time.  *Increase no*: Don’t give up. Stay tuned for more tips. |
| 11. Demonstrate/ show interest in the person | Provide statements of interest and curiosity about the patient’s thoughts or perceptions, personal history, and social context, when communicating about psychosocial pain treatments. | Do you sometimes find yourself feeling negative feelings like guilt, frustration, and sadness when you are in pain? You are not alone! The good news is there's a treatment that can help you manage pain by confronting these feelings head-on. |
| 12. Use empathic listening | *Most relevant to SMS text messaging:* Demonstrate attentiveness to the patient’s responses to messages about psychosocial treatments. Wait for the patient to respond to automated messages to provide additional, relevant guidance. | Interactive Question: So, how did you do making changes in your sleep routine? Type *Sleep* and *Very well, Okay, Not very well*.  *Very well*: Great, keep up the good work. Over time, these changes will help manage your pain.  *Okay*: Keep working on making these changes. Over time, these changes will help manage your pain. If you need extra support, try CBT-i Coach.^1^  *Not very well*: Sorry to hear that. If you need extra support, try CBT-i Coach. You could also reach out to your VA care team to discuss your sleep routine and goals. |
| 13. Providing opportunities for ongoing support | Offer the patient concrete points of contact and/or next steps to learn about psychosocial pain treatments. | As a Veteran, you may have access to My HealtheVet, which is a helpful tool you can use to communicate with your health care team. You can even send secure messages to your health care team about non-emergency information or other questions. |
| 14. Prompt identification and seek available social support | Prompt the patient to identify sources of support for discussions about pain management. | Consider if Mindfulness-Based Stress Reduction for chronic pain is right for you by discussing with your family members, peers, and local VA care team. |
| **Competence-support techniques** | | |
| 15. Address obstacles for change | Prompt the patient to identify likely barriers to change in pain management routines and offer ways to overcome them. | Another tip for improving your sleep is to keep a regular routine. Try to go to bed and wake up around the same time each day. Consider avoiding naps because they can interfere with your sleep schedule. |
| 16. Clarify expectations | Prompt the patient to think about their own expectations in terms of changing their pain management routine, both in terms of process for seeking care and pain-related outcomes. | Although it may be unrealistic to expect that chronic pain will disappear, the approaches learned in Cognitive Behavioral Therapy may help you do more of the things you would like to do by reacting to pain in a way that makes it feel less overwhelming. |
| 17. Assist in setting optimal challenge | Assist the patient to identify goals that are realistic, meaningfully challenging, and achievable. | While pacing your activities can take more time, you will get more done and have less pain. |
| 18. Offer constructive, clear, and relevant feedback | *Most relevant to SMS text messaging:* Provide relevant, tailored, non-evaluative feedback on patient responses to communications about psychosocial pain treatments. | Interactive Question: Tell me how you did with taking your mind off your pain. Type *Calm* and *Very well*, *Okay*, or *Not very well*.  *Very well*: Great, keep up the good work. Over time, these changes will help manage your pain.  *Okay*: Keep working on making these changes. Over time, these changes will help manage your pain.  *Not very well*: Don’t give up, and if you need extra support, remember to try Mindfulness Coach.^2^ |
| 19. Help develop a clear and concrete plan of action | Provide a summary of concrete steps to work towards the patient changing their current approach to pain management. | Consider dividing activities into smaller parts. Instead of doing everything at once, spread things out over the week or take frequent breaks. |
| 20. Promote self-monitoring | Provide examples to help the patient understand how they currently manage pain. | Think of yourself as a filter. When a feeling comes to the surface, we can choose either to let it pass through us like a liquid or bog us down by letting it linger and solidify. |
| 21. Explore ways of dealing with pressure | Provide information to manage and limit pressures that would undermine the patient’s competence or capacity to seek information about psychosocial pain treatments. | If it helps you feel better, you could take a moment to think about your activities and plan ahead to avoid doing too much or too little. This can help reduce pain flare-ups. |

^1^CBT-i Coach is a VA-developed app to support veterans and others engaged in Cognitive Behavioral Therapy for Insomnia with a health provider, or who have experienced symptoms of Insomnia and would like to improve sleep habits on their own. A link to download the app was provided in the message (<https://mobile.va.gov/app/cbt-i-coach>).

^2^Mindfulness Coach is a VA-developed app designed for veterans and others to learn about practicing mindfulness. A link to download the app was provided in the message (<https://mobile.va.gov/app/mindfulness-coach>).
