# Supplementary material for: Designing Patient-Friendly Messages: Tutorial on Applying Human-Centered, Self-Determination Theory With AI Considerations
Source: J Med Internet Res. 2025 Oct 17;27:e78173. doi: 10.2196/78173 (PMC12579294; doi:10.2196/78173)
Supplement: Multimedia Appendix 2 [file jmir_v27i1e78173_app2.pptx]

## Slide 1
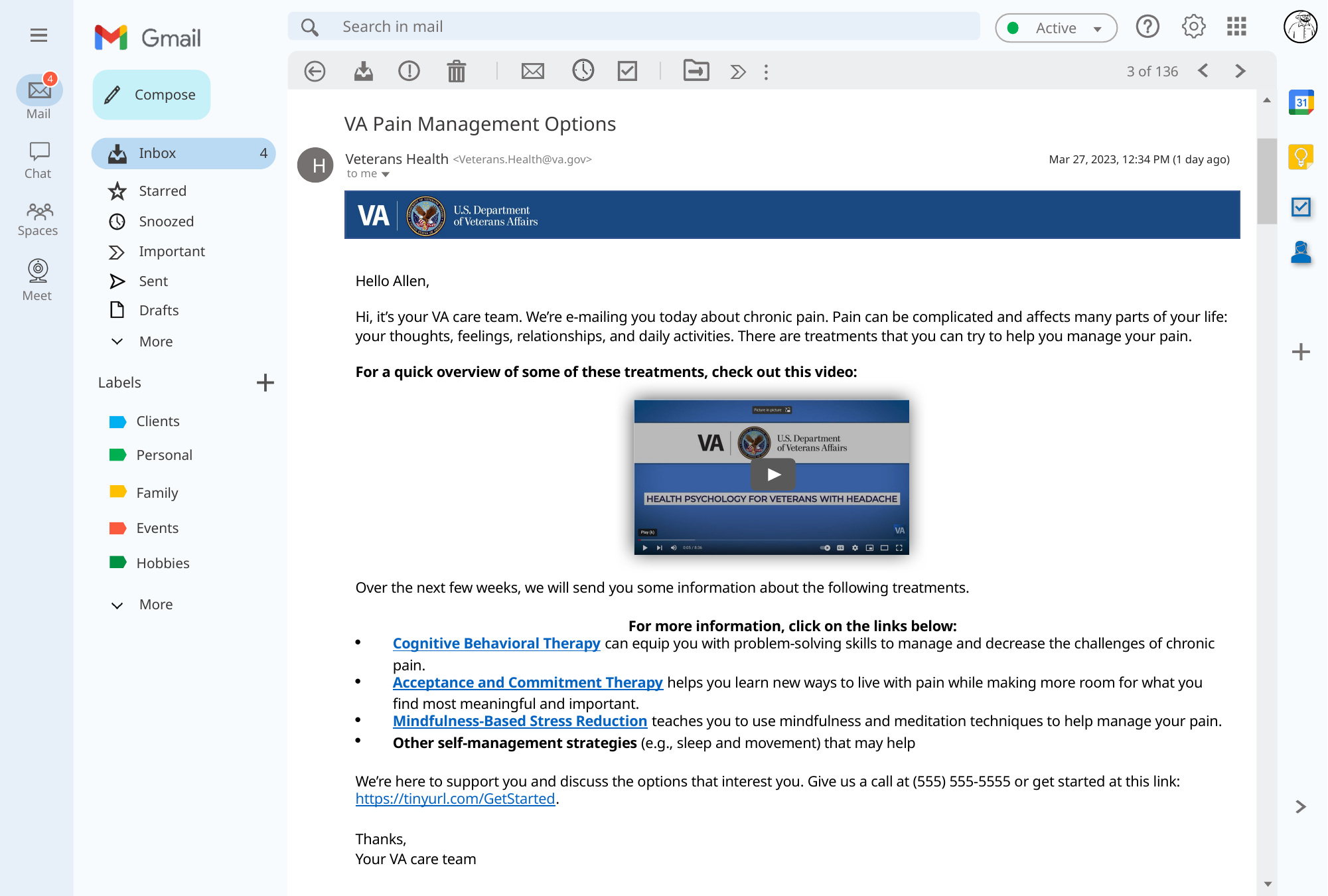

Search in mail
Compose
Inbox 4
Starred
Snoozed
Important
Sent
Drafts
More
…
Active
3 of 136
4
Mail
Chat
Spaces
Meet
Labels
Clients
Personal
Family
Events
Hobbies
More
VA Pain Management Options
Veterans Health
H
<Veterans.Health@va.gov>
to me
Mar 27, 2023, 12:34 PM (1 day ago)
Hello Allen,
Hi, it’s your VA care team. We’re e-mailing you today about chronic pain. Pain can be complicated and affects many parts of your life: your thoughts, feelings, relationships, and daily activities. There are treatments that you can try to help you manage your pain.
For a quick overview of some of these treatments, check out this video:
Over the next few weeks, we will send you some information about the following treatments.
For more information, click on the links below:
Cognitive Behavioral Therapy can equip you with problem-solving skills to manage and decrease the challenges of chronic pain.
Acceptance and Commitment Therapy helps you learn new ways to live with pain while making more room for what you find most meaningful and important.
Mindfulness-Based Stress Reduction teaches you to use mindfulness and meditation techniques to help manage your pain.
Other self-management strategies (e.g., sleep and movement) that may help
We’re here to support you and discuss the options that interest you. Give us a call at (555) 555-5555 or get started at this link: https://tinyurl.com/GetStarted.
Thanks,
Your VA care team

## Slide 2
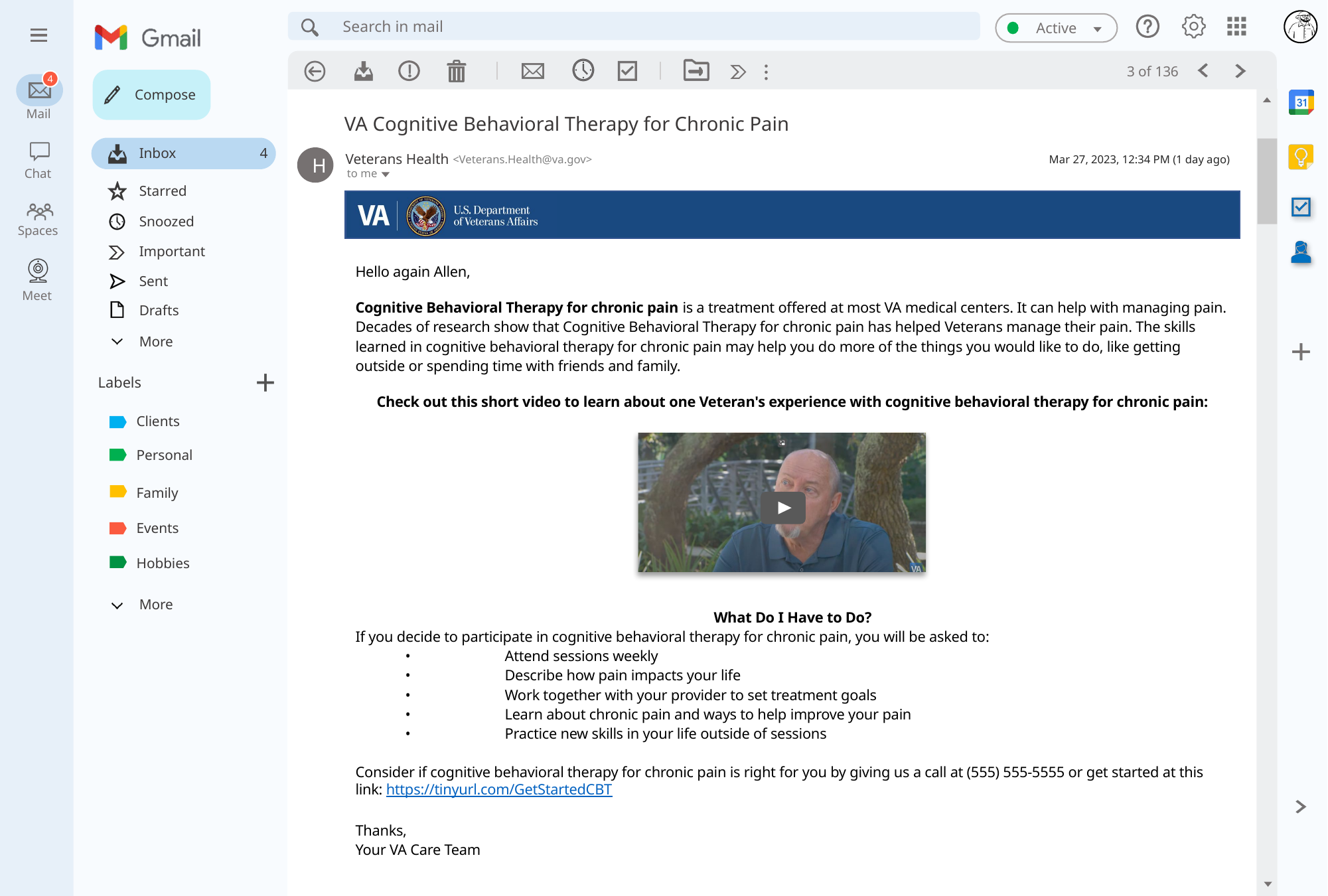

Search in mail
Compose
Inbox 4
Starred
Snoozed
Important
Sent
Drafts
More
…
Active
3 of 136
4
Mail
Chat
Spaces
Meet
Labels
Clients
Personal
Family
Events
Hobbies
More
VA Cognitive Behavioral Therapy for Chronic Pain
Veterans Health
H
<Veterans.Health@va.gov>
to me
Mar 27, 2023, 12:34 PM (1 day ago)
Hello again Allen,
Cognitive Behavioral Therapy for chronic pain is a treatment offered at most VA medical centers. It can help with managing pain. Decades of research show that Cognitive Behavioral Therapy for chronic pain has helped Veterans manage their pain. The skills learned in cognitive behavioral therapy for chronic pain may help you do more of the things you would like to do, like getting outside or spending time with friends and family.
Check out this short video to learn about one Veteran's experience with cognitive behavioral therapy for chronic pain:
What Do I Have to Do?
If you decide to participate in cognitive behavioral therapy for chronic pain, you will be asked to:
•	Attend sessions weekly
•	Describe how pain impacts your life
•	Work together with your provider to set treatment goals
•	Learn about chronic pain and ways to help improve your pain
•	Practice new skills in your life outside of sessions
Consider if cognitive behavioral therapy for chronic pain is right for you by giving us a call at (555) 555-5555 or get started at this link: https://tinyurl.com/GetStartedCBT
Thanks,
Your VA Care Team

## Slide 3
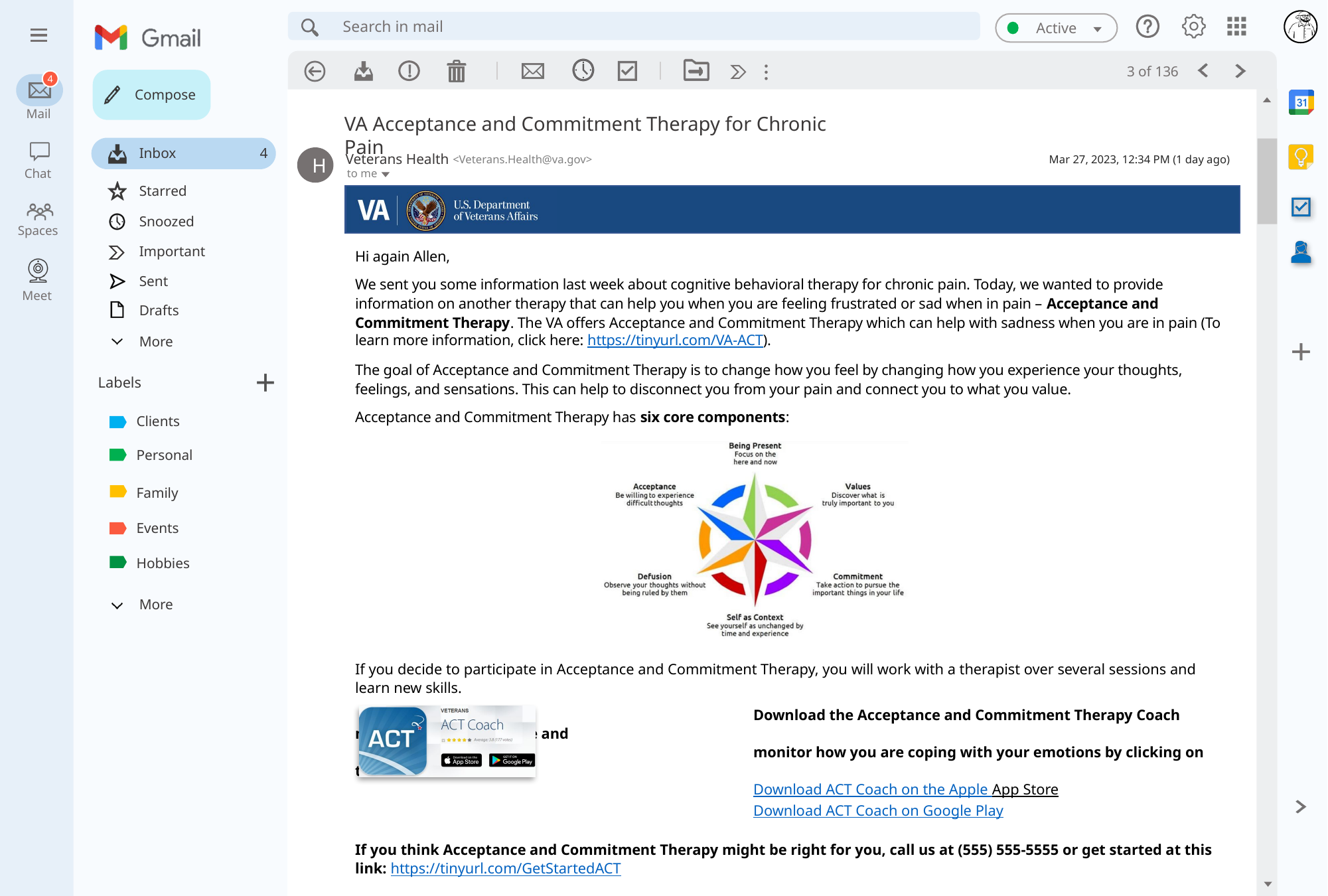

Search in mail
Compose
Inbox 4
Starred
Snoozed
Important
Sent
Drafts
More
…
Active
3 of 136
4
Mail
Chat
Spaces
Meet
Labels
Clients
Personal
Family
Events
Hobbies
More
VA Acceptance and Commitment Therapy for Chronic Pain
Veterans Health
H
<Veterans.Health@va.gov>
to me
Mar 27, 2023, 12:34 PM (1 day ago)
Hi again Allen,
We sent you some information last week about cognitive behavioral therapy for chronic pain. Today, we wanted to provide information on another therapy that can help you when you are feeling frustrated or sad when in pain – Acceptance and Commitment Therapy. The VA offers Acceptance and Commitment Therapy which can help with sadness when you are in pain (To learn more information, click here: https://tinyurl.com/VA-ACT).
The goal of Acceptance and Commitment Therapy is to change how you feel by changing how you experience your thoughts, feelings, and sensations. This can help to disconnect you from your pain and connect you to what you value.
Acceptance and Commitment Therapy has six core components:
If you decide to participate in Acceptance and Commitment Therapy, you will work with a therapist over several sessions and learn new skills.
				Download the Acceptance and Commitment Therapy Coach mobile app to learn more and
				monitor how you are coping with your emotions by clicking on these links:
				Download ACT Coach on the Apple App Store
				Download ACT Coach on Google Play
If you think Acceptance and Commitment Therapy might be right for you, call us at (555) 555-5555 or get started at this link: https://tinyurl.com/GetStartedACT
Thanks,
Your VA care team

## Slide 4
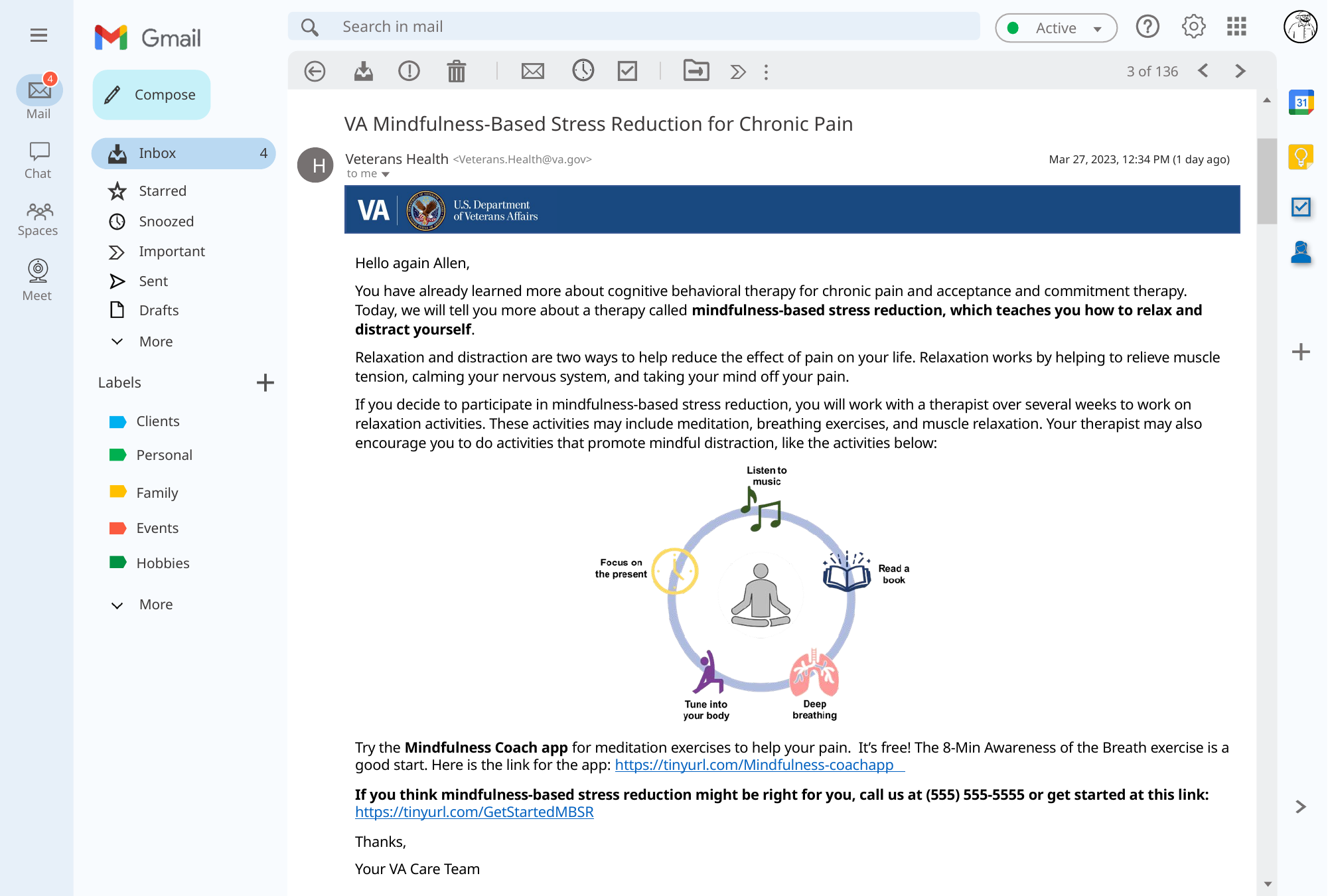

Search in mail
Compose
Inbox 4
Starred
Snoozed
Important
Sent
Drafts
More
…
Active
3 of 136
4
Mail
Chat
Spaces
Meet
Labels
Clients
Personal
Family
Events
Hobbies
More
VA Mindfulness-Based Stress Reduction for Chronic Pain
Veterans Health
H
<Veterans.Health@va.gov>
to me
Mar 27, 2023, 12:34 PM (1 day ago)
Hello again Allen,
You have already learned more about cognitive behavioral therapy for chronic pain and acceptance and commitment therapy. Today, we will tell you more about a therapy called mindfulness-based stress reduction, which teaches you how to relax and distract yourself.
Relaxation and distraction are two ways to help reduce the effect of pain on your life. Relaxation works by helping to relieve muscle tension, calming your nervous system, and taking your mind off your pain.
If you decide to participate in mindfulness-based stress reduction, you will work with a therapist over several weeks to work on relaxation activities. These activities may include meditation, breathing exercises, and muscle relaxation. Your therapist may also encourage you to do activities that promote mindful distraction, like the activities below:
Try the Mindfulness Coach app for meditation exercises to help your pain. It’s free! The 8-Min Awareness of the Breath exercise is a good start. Here is the link for the app: https://tinyurl.com/Mindfulness-coachapp
If you think mindfulness-based stress reduction might be right for you, call us at (555) 555-5555 or get started at this link: https://tinyurl.com/GetStartedMBSR
Thanks,
Your VA Care Team

## Slide 5
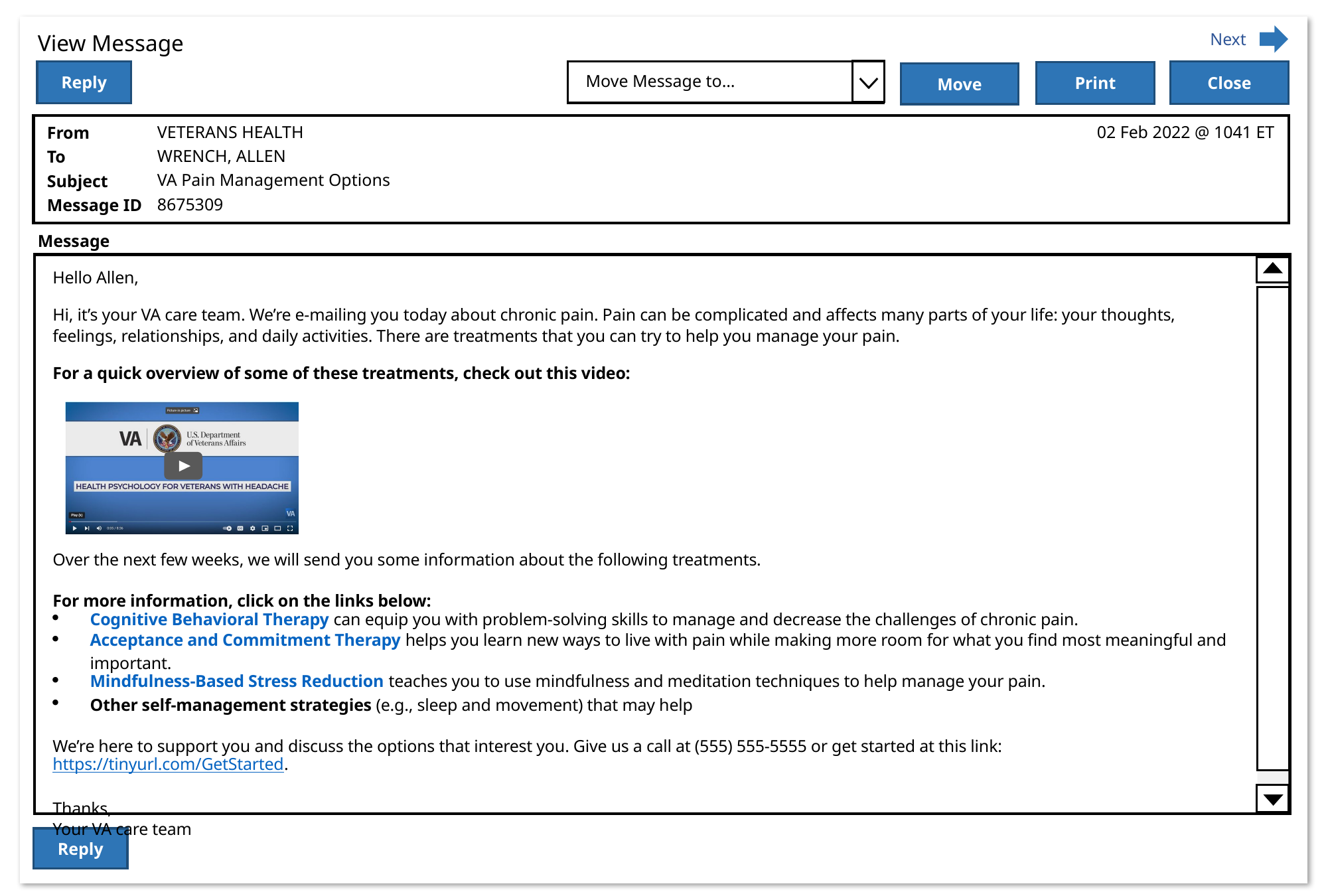

Next
View Message
Close
Reply
Print
Move
Move Message to…
02 Feb 2022 @ 1041 ET
VETERANS HEALTH
WRENCH, ALLEN
VA Pain Management Options
8675309
From
To
Subject
Message ID
Message
Hello Allen,
Hi, it’s your VA care team. We’re e-mailing you today about chronic pain. Pain can be complicated and affects many parts of your life: your thoughts, feelings, relationships, and daily activities. There are treatments that you can try to help you manage your pain.
For a quick overview of some of these treatments, check out this video:
Over the next few weeks, we will send you some information about the following treatments.
For more information, click on the links below:
Cognitive Behavioral Therapy can equip you with problem-solving skills to manage and decrease the challenges of chronic pain.
Acceptance and Commitment Therapy helps you learn new ways to live with pain while making more room for what you find most meaningful and important.
Mindfulness-Based Stress Reduction teaches you to use mindfulness and meditation techniques to help manage your pain.
Other self-management strategies (e.g., sleep and movement) that may help
We’re here to support you and discuss the options that interest you. Give us a call at (555) 555-5555 or get started at this link: https://tinyurl.com/GetStarted.
Thanks,
Your VA care team
Reply

## Slide 6
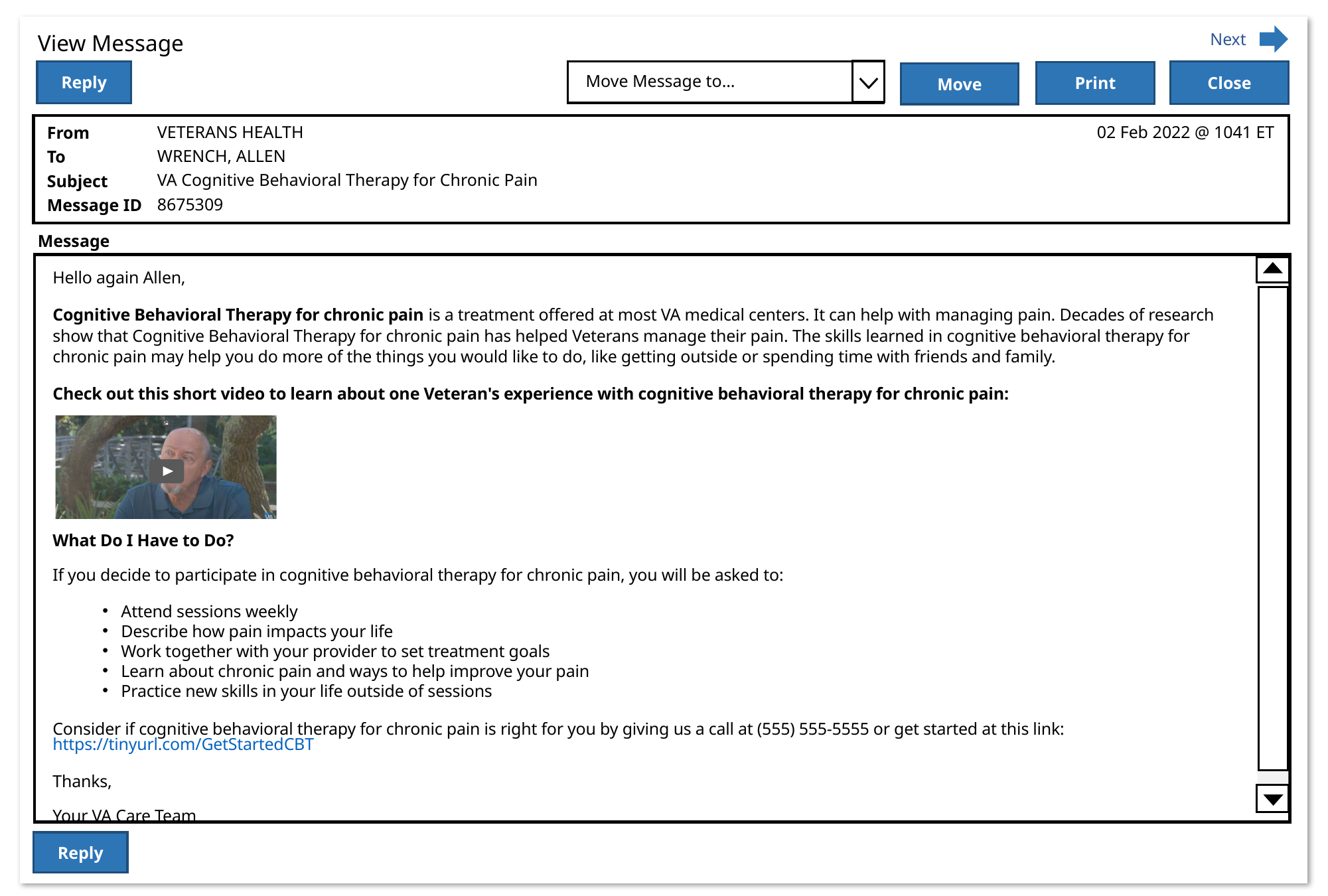

Next
View Message
Close
Reply
Print
Move
Move Message to…
02 Feb 2022 @ 1041 ET
VETERANS HEALTH
WRENCH, ALLEN
VA Cognitive Behavioral Therapy for Chronic Pain
8675309
From
To
Subject
Message ID
Message
Hello again Allen,
Cognitive Behavioral Therapy for chronic pain is a treatment offered at most VA medical centers. It can help with managing pain. Decades of research show that Cognitive Behavioral Therapy for chronic pain has helped Veterans manage their pain. The skills learned in cognitive behavioral therapy for chronic pain may help you do more of the things you would like to do, like getting outside or spending time with friends and family.
Check out this short video to learn about one Veteran's experience with cognitive behavioral therapy for chronic pain:
What Do I Have to Do?
If you decide to participate in cognitive behavioral therapy for chronic pain, you will be asked to:
Attend sessions weekly
Describe how pain impacts your life
Work together with your provider to set treatment goals
Learn about chronic pain and ways to help improve your pain
Practice new skills in your life outside of sessions
Consider if cognitive behavioral therapy for chronic pain is right for you by giving us a call at (555) 555-5555 or get started at this link: https://tinyurl.com/GetStartedCBT
Thanks,
Your VA Care Team
Reply

## Slide 7
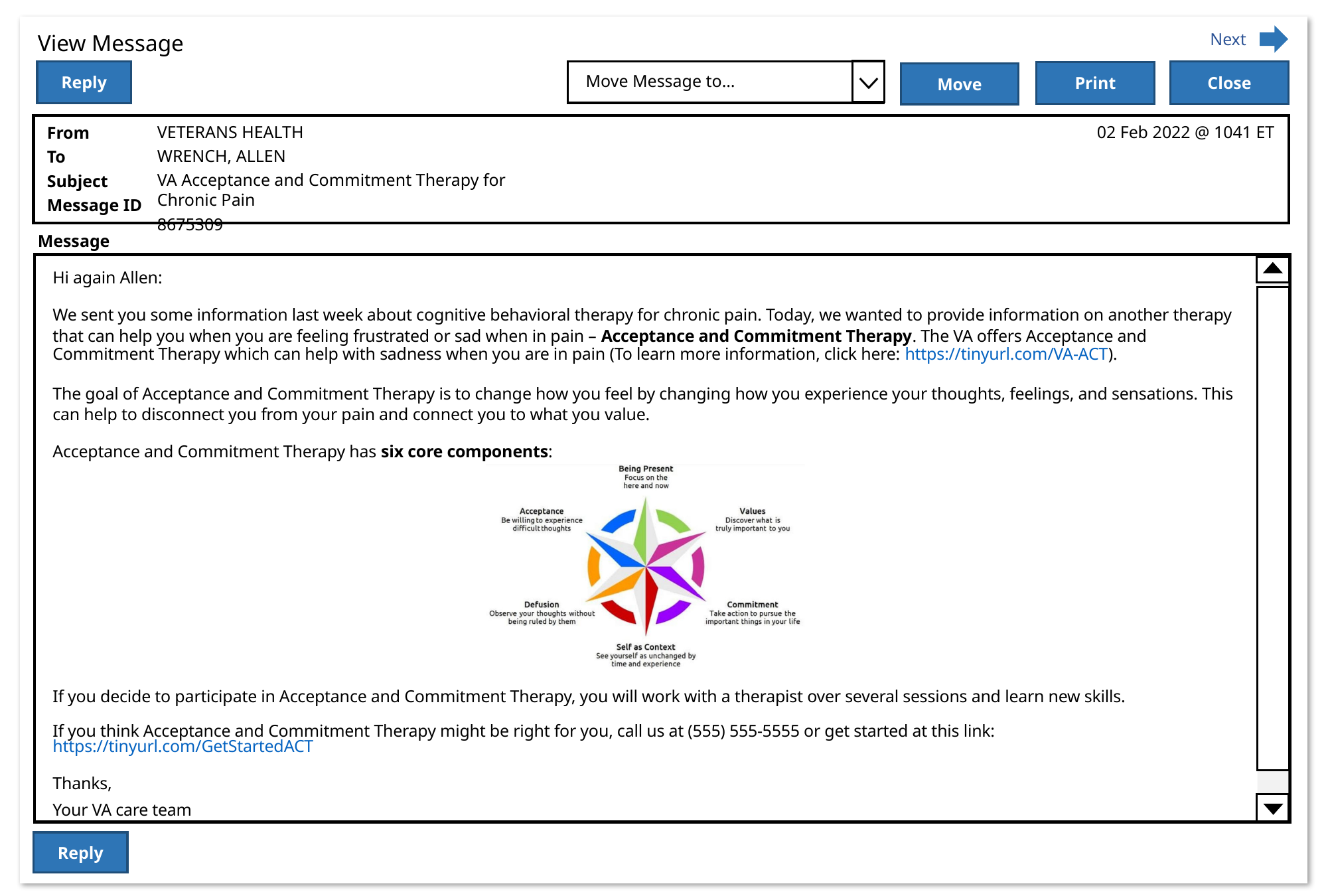

Next
View Message
Close
Reply
Print
Move
Move Message to…
02 Feb 2022 @ 1041 ET
VETERANS HEALTH
WRENCH, ALLEN
VA Acceptance and Commitment Therapy for Chronic Pain
8675309
From
To
Subject
Message ID
Message
Hi again Allen:
We sent you some information last week about cognitive behavioral therapy for chronic pain. Today, we wanted to provide information on another therapy that can help you when you are feeling frustrated or sad when in pain – Acceptance and Commitment Therapy. The VA offers Acceptance and Commitment Therapy which can help with sadness when you are in pain (To learn more information, click here: https://tinyurl.com/VA-ACT).
The goal of Acceptance and Commitment Therapy is to change how you feel by changing how you experience your thoughts, feelings, and sensations. This can help to disconnect you from your pain and connect you to what you value.
Acceptance and Commitment Therapy has six core components:
If you decide to participate in Acceptance and Commitment Therapy, you will work with a therapist over several sessions and learn new skills.
If you think Acceptance and Commitment Therapy might be right for you, call us at (555) 555-5555 or get started at this link: https://tinyurl.com/GetStartedACT
Thanks,
Your VA care team
Reply

## Slide 8
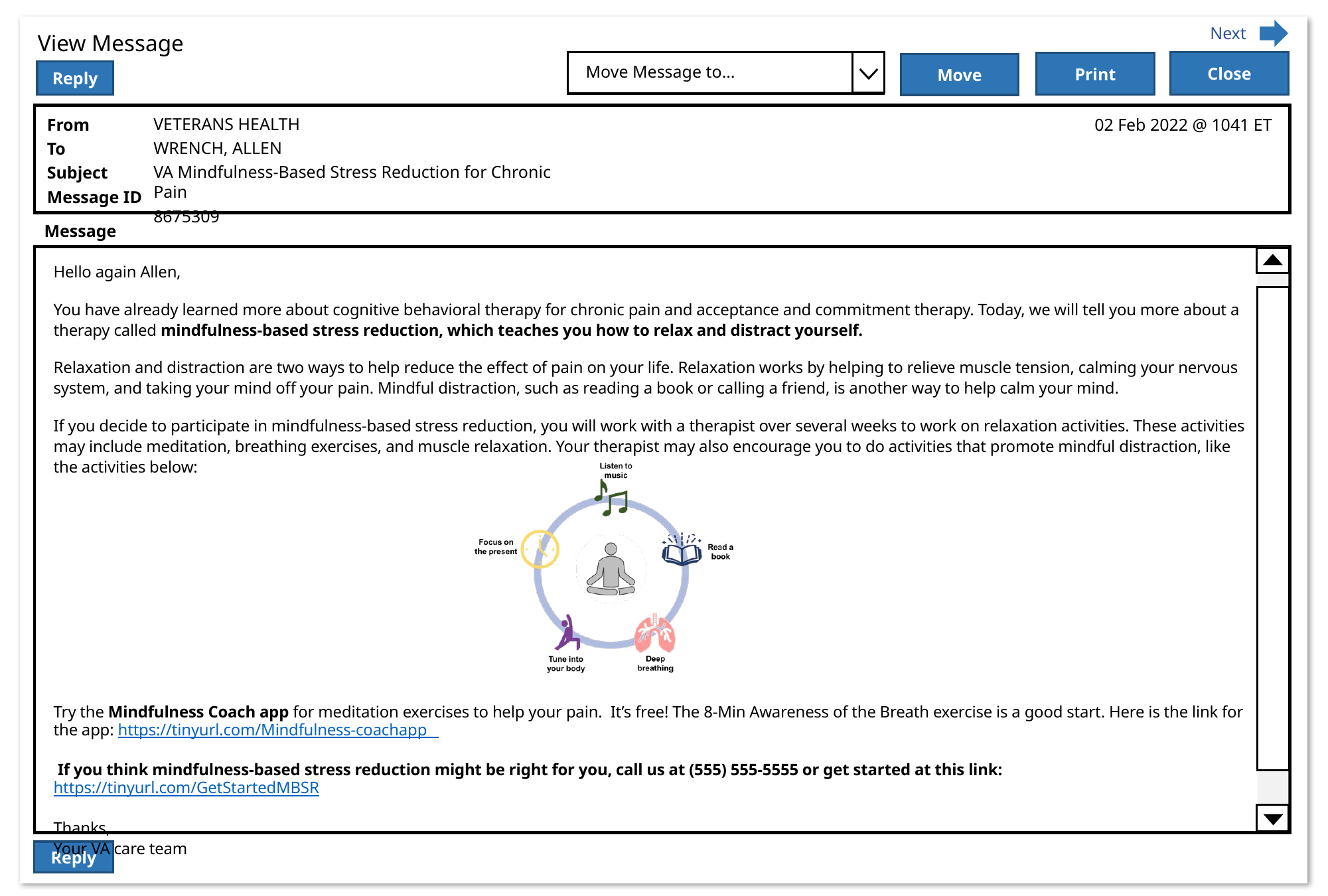

Next
View Message
Close
Print
Move
Move Message to…
Reply
VETERANS HEALTH
WRENCH, ALLEN
VA Mindfulness-Based Stress Reduction for Chronic Pain
8675309
From
To
Subject
Message ID
02 Feb 2022 @ 1041 ET
Message
Hello again Allen,
You have already learned more about cognitive behavioral therapy for chronic pain and acceptance and commitment therapy. Today, we will tell you more about a therapy called mindfulness-based stress reduction, which teaches you how to relax and distract yourself.
Relaxation and distraction are two ways to help reduce the effect of pain on your life. Relaxation works by helping to relieve muscle tension, calming your nervous system, and taking your mind off your pain. Mindful distraction, such as reading a book or calling a friend, is another way to help calm your mind.
If you decide to participate in mindfulness-based stress reduction, you will work with a therapist over several weeks to work on relaxation activities. These activities may include meditation, breathing exercises, and muscle relaxation. Your therapist may also encourage you to do activities that promote mindful distraction, like the activities below:
Try the Mindfulness Coach app for meditation exercises to help your pain. It’s free! The 8-Min Awareness of the Breath exercise is a good start. Here is the link for the app: https://tinyurl.com/Mindfulness-coachapp
 If you think mindfulness-based stress reduction might be right for you, call us at (555) 555-5555 or get started at this link: https://tinyurl.com/GetStartedMBSR
Thanks,
Your VA care team
Reply

## Slide 9
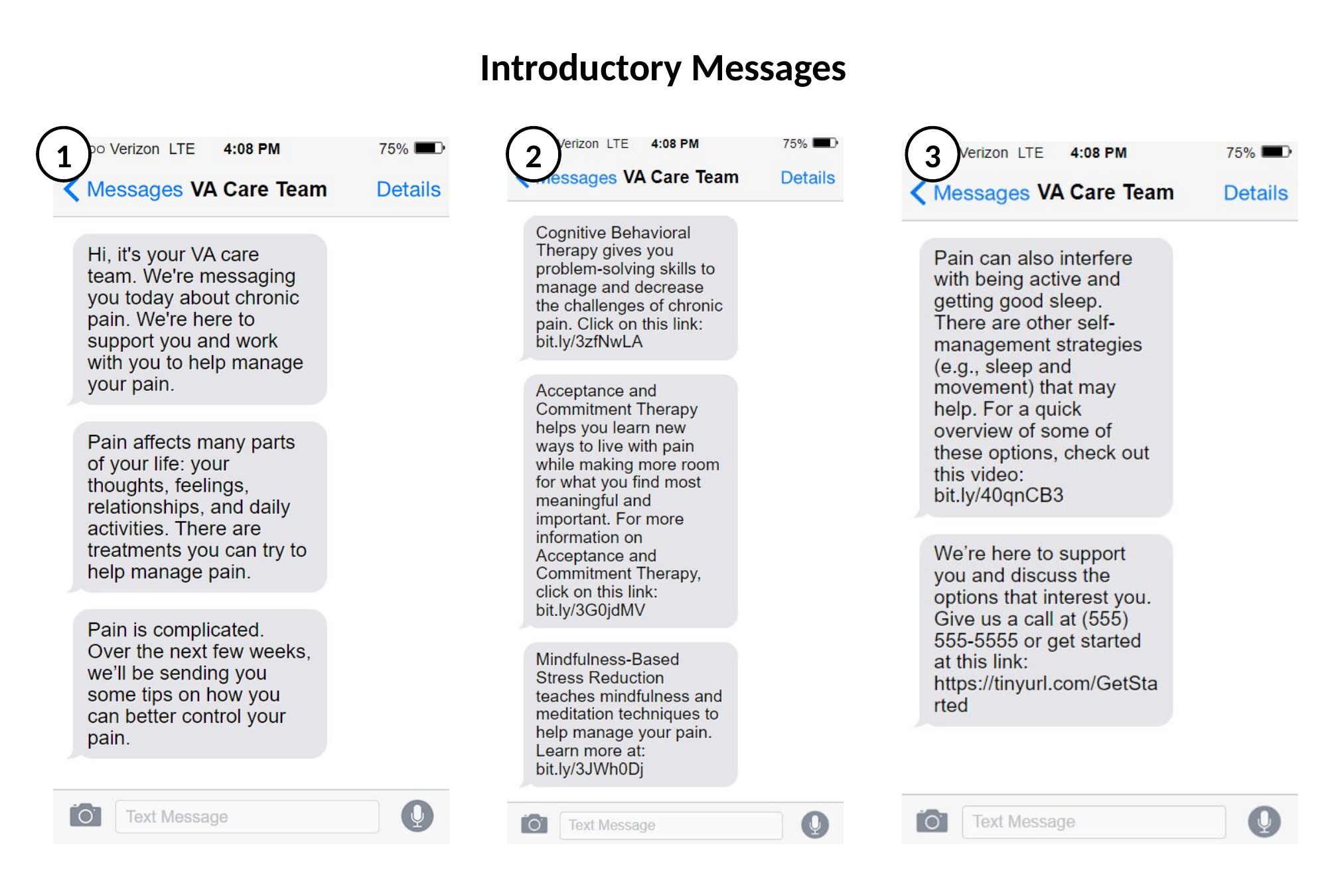

Introductory Messages
3
1
2

## Slide 10
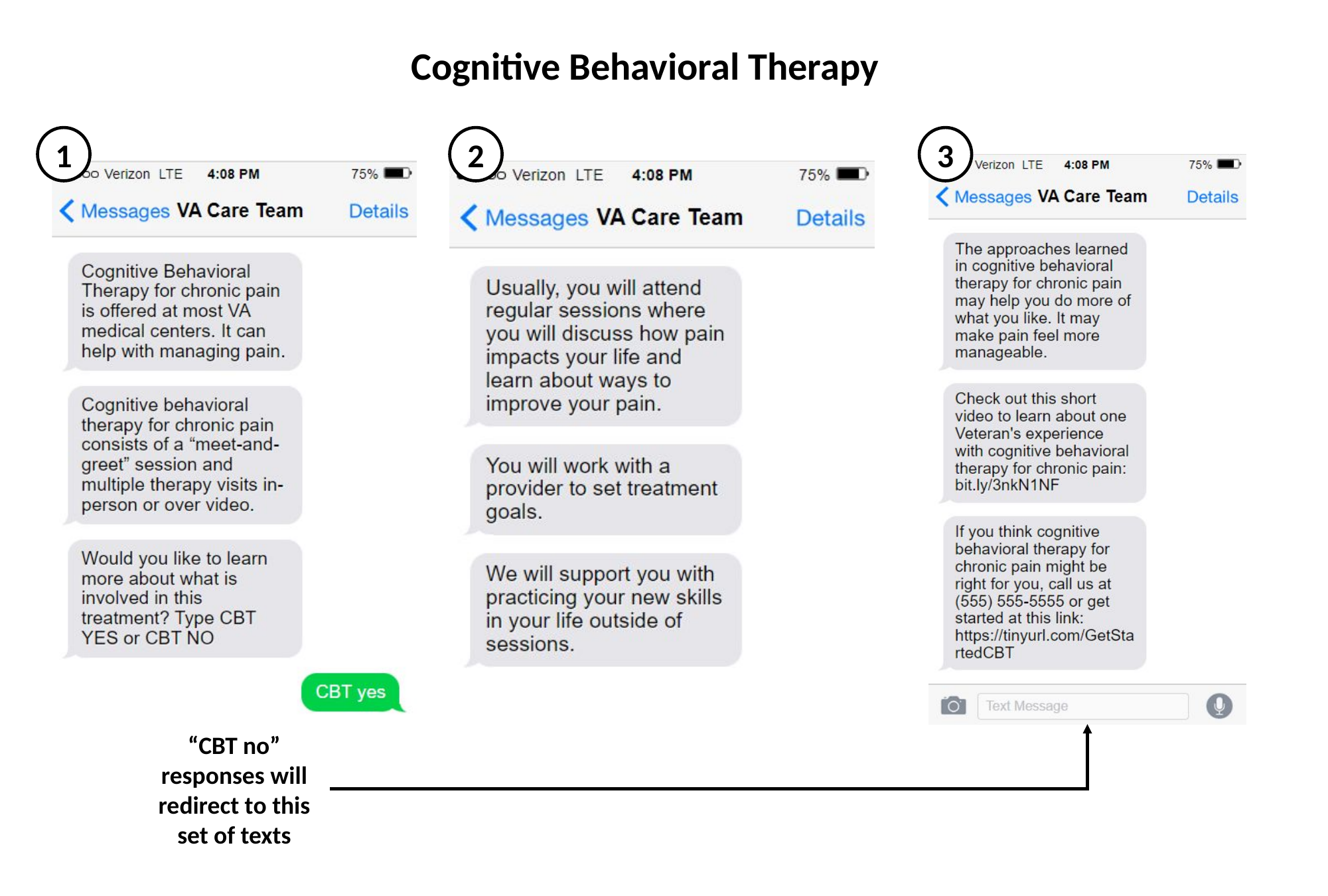

Cognitive Behavioral Therapy
1
2
3
“CBT no” responses will redirect to this set of texts

## Slide 11
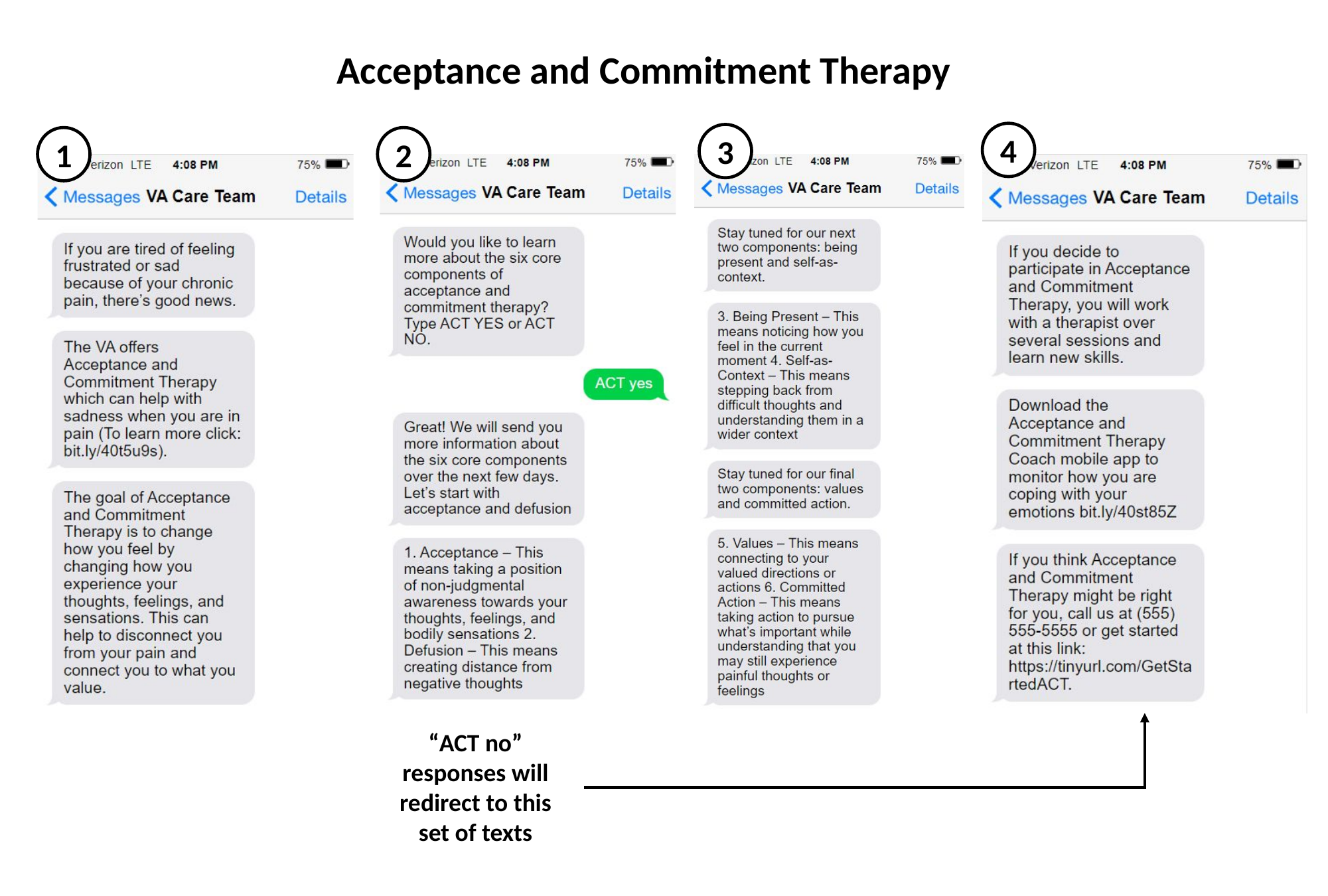

Acceptance and Commitment Therapy
4
3
1
2
“ACT no” responses will redirect to this set of texts

## Slide 12
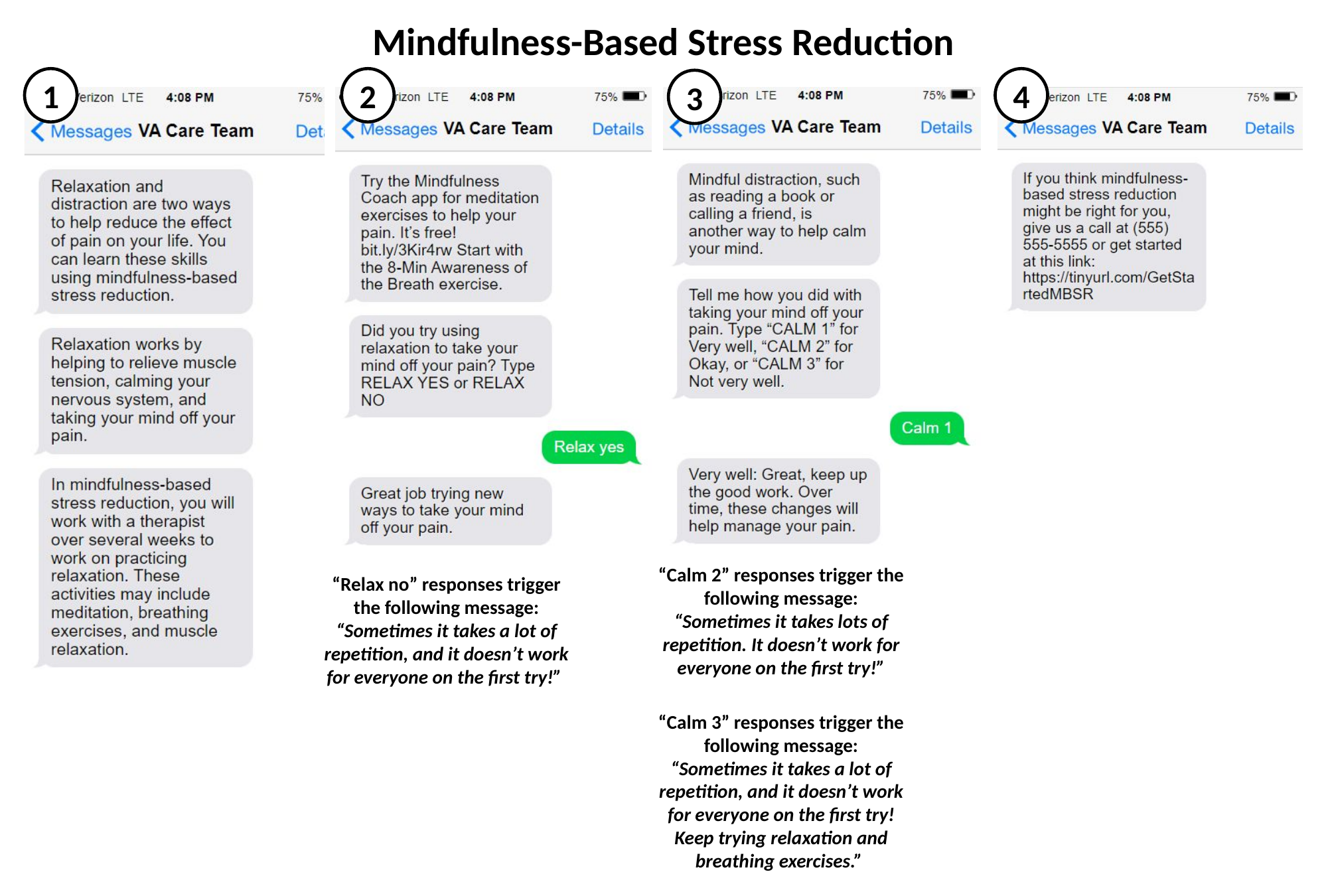

Mindfulness-Based Stress Reduction
1
2
4
3
“Calm 2” responses trigger the following message: “Sometimes it takes lots of repetition. It doesn’t work for everyone on the first try!”
“Relax no” responses trigger the following message: “Sometimes it takes a lot of repetition, and it doesn’t work for everyone on the first try!”
“Calm 3” responses trigger the following message: “Sometimes it takes a lot of repetition, and it doesn’t work for everyone on the first try! Keep trying relaxation and breathing exercises.”
